# Supplementary material for: Geobarrettin D, a Rare Herbipoline-Containing 6-Bromoindole Alkaloid from Geodia barretti
Source: Molecules. 2023 Mar 24;28(7):2937. doi: 10.3390/molecules28072937 (PMC10095911; doi:10.3390/molecules28072937)
Supplement: Supplementary file 1 [file molecules-28-02937-s001.zip › molecules-2237853-supplementary.pdf]

Supporting Information

Geobarrettin D, a Rare Herbipoline-Containing 6-Bromoindole Alkaloid from *Geodia barretti*

Xiaxia Di [1,2,†], Ingibjorg Hardardottir [2,3], Jona Freysdottir [2,3], Dongdong Wang [4], Kirk R. Gustafson [4], Sesselja Omarsdottir [1,*] and Tadeusz F. Molinski [5,*]

[1] Faculty of Pharmaceutical Sciences, University of Iceland, Hagi, Hofsvallagata 53, IS-107 Reykjavik, Iceland
[2] Department of Immunology, Landspitali—The National University Hospital of Iceland, IS-101 Reykjavik, Iceland
[3] Faculty of Medicine, Biomedical Center, University of Iceland, Vatnsmyrarvegur 16, IS-101 Reykjavik, Iceland
[4] Molecular Targets Program, Center for Cancer Research, National Cancer Institute, Frederick, MD 21702, USA
[5] Department of Chemistry and Biochemistry, Skaggs School of Pharmacy and Pharmaceutical Sciences, University of California, San Diego, CA 92093, USA
* Correspondence: sesselo@hi.is (S.O.); tmolinski@ucsd.edu (T.F.M.); Tel.: +354-842-4514 (S.O.); +1-858-534-7115 (T.F.M.)
† Current address: Departments of Biochemistry and Biomedical Sciences & Chemistry and Chemical Biology, M. G. DeGroote Institute for Infectious Disease Research, McMaster University, Hamilton, ON L8S 4K1, Canada

Figure S1. ^1H NMR spectrum of geobarrettin D (**1**), recorded in CD_3OD, 600 MHz

Figure S2. ^{13}C NMR spectrum of geobarrettin D (**1**), recorded in CD_3OD, 150 MHz

Figure S3. DEPT-135 NMR spectrum of geobarrettin D (**1**)

Figure S4. HSQC spectrum of geobarrettin D (**1**)

Figure S5. HMBC spectrum of geobarrettin D (**1**)

Figure S6. COSY spectrum of geobarrettin D (**1**)

Figure S7. ^1H NMR spectrum of geobarrettin D (**1**), recorded in D_2O/H_2O 10/90, 600 MHz

Figure S8. ^1H-^{15}N HSQC spectrum of geobarrettin D (**1**), recorded in D_2O/H_2O 10/90

Figure S9. ^1H-^{15}N HMBC spectrum of geobarrettin D (**1**), recorded in D_2O/H_2O 10/90

Figure S10. ESI spectrum of geobarrettin D (1)

Figure S11. IR spectrum of geobarrettin D (**1**)

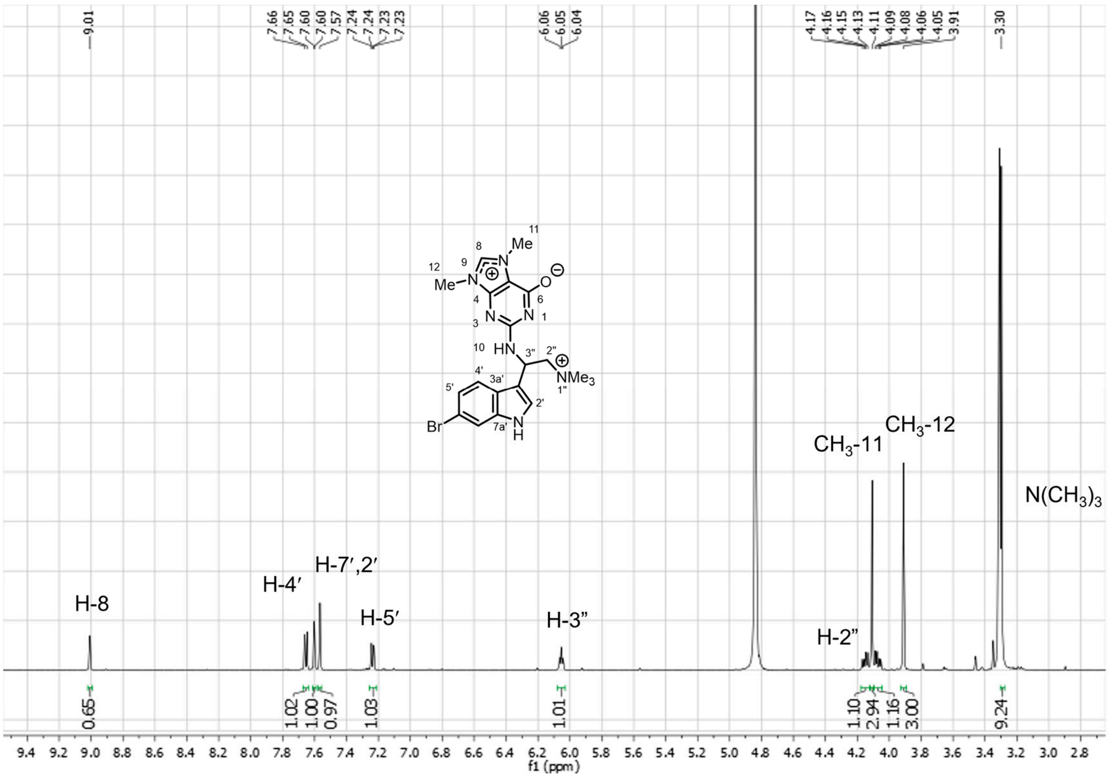

Figure S1. ^{1}H NMR spectrum of geobarrettin D (**1**), recorded in CD3OD, 600 MHz.

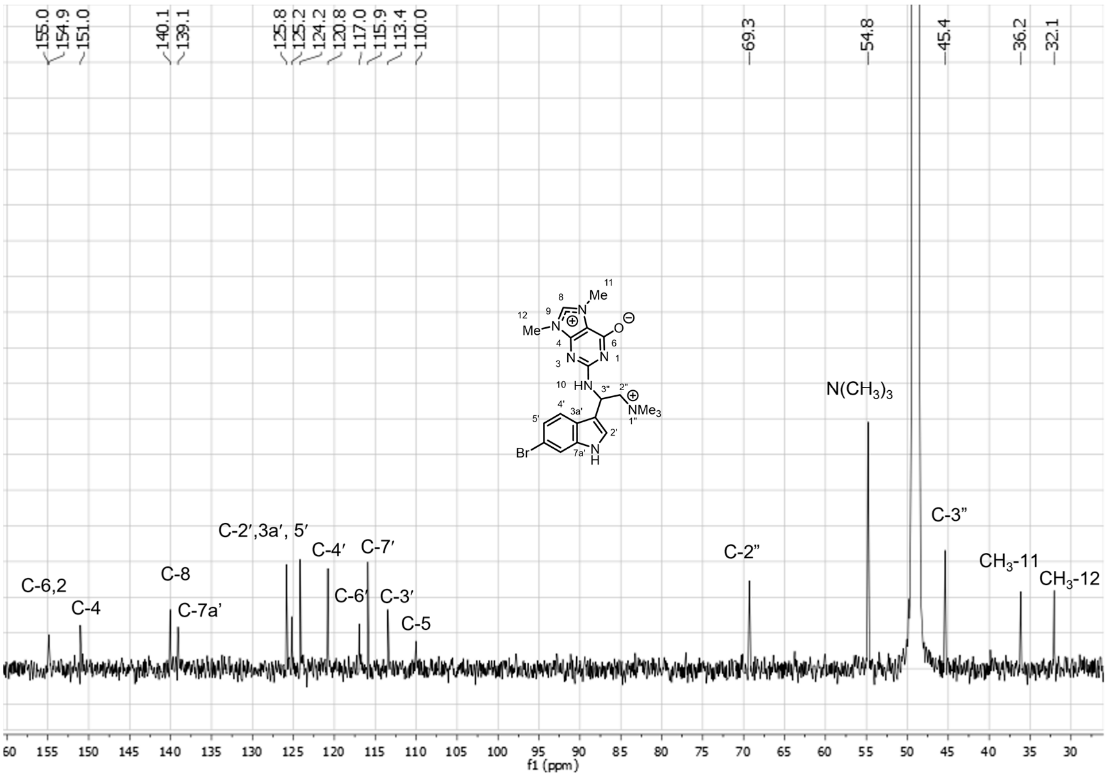

Figure S2. ^{13}C NMR spectrum of geobarrettin D (**1**), recorded in CD3OD, 150 MHz.

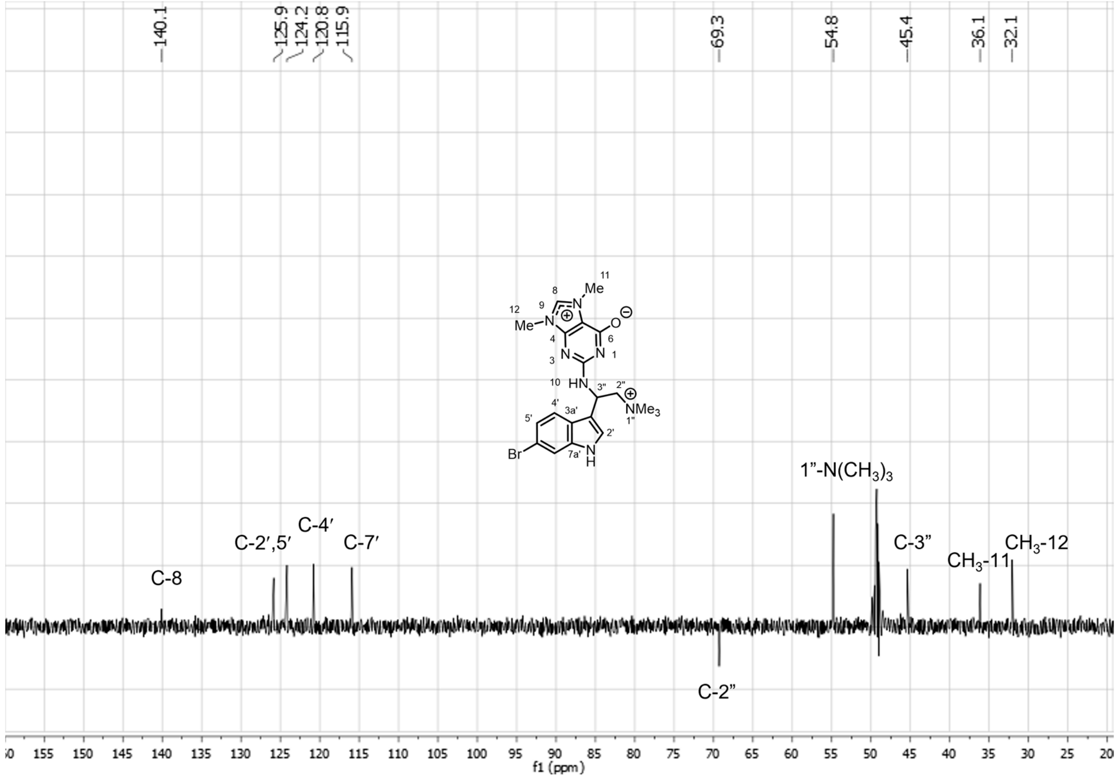

Figure S3. DEPT-135 NMR spectrum of geobarrettin D (**1**).

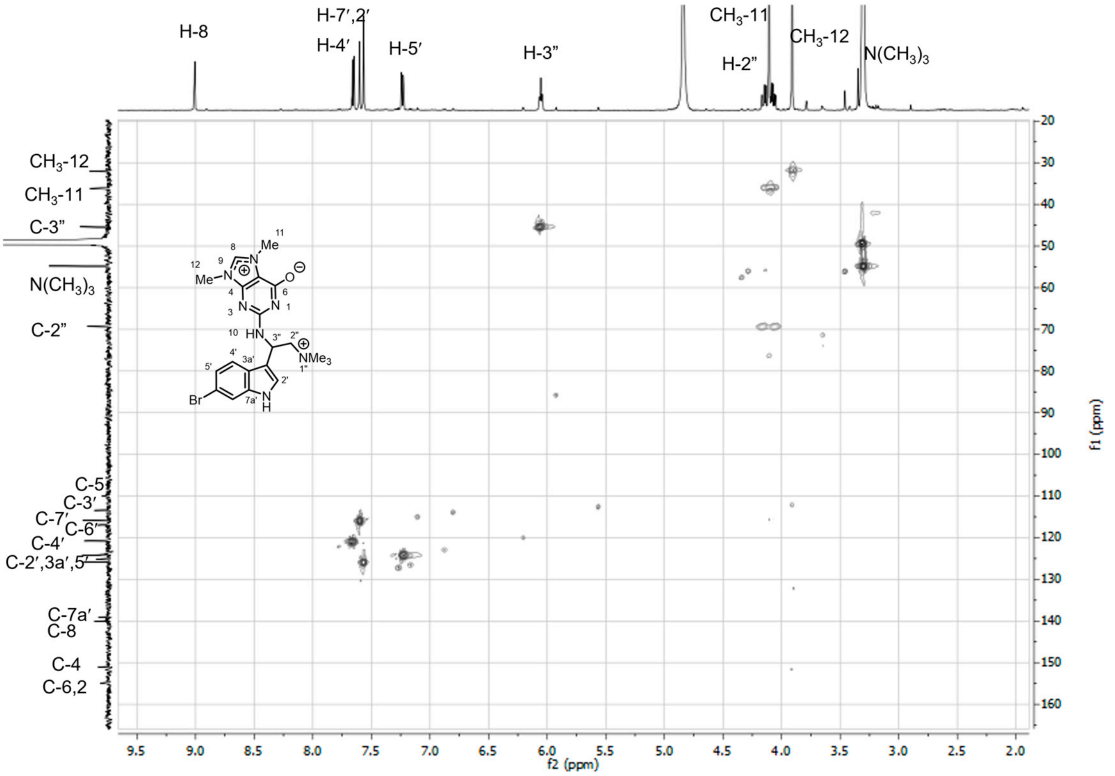

Figure S4. HSQC spectrum of geobarrettin D (**1**).

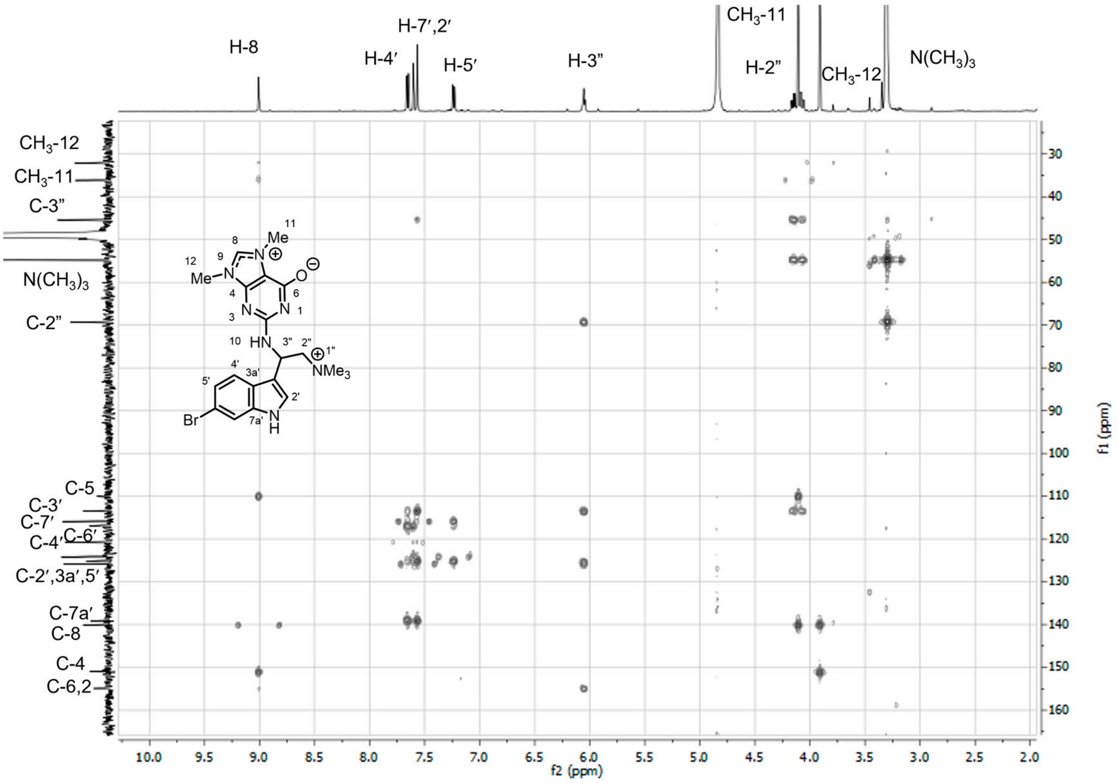

Figure S5. HMBC spectrum of geobarrettin D (1).

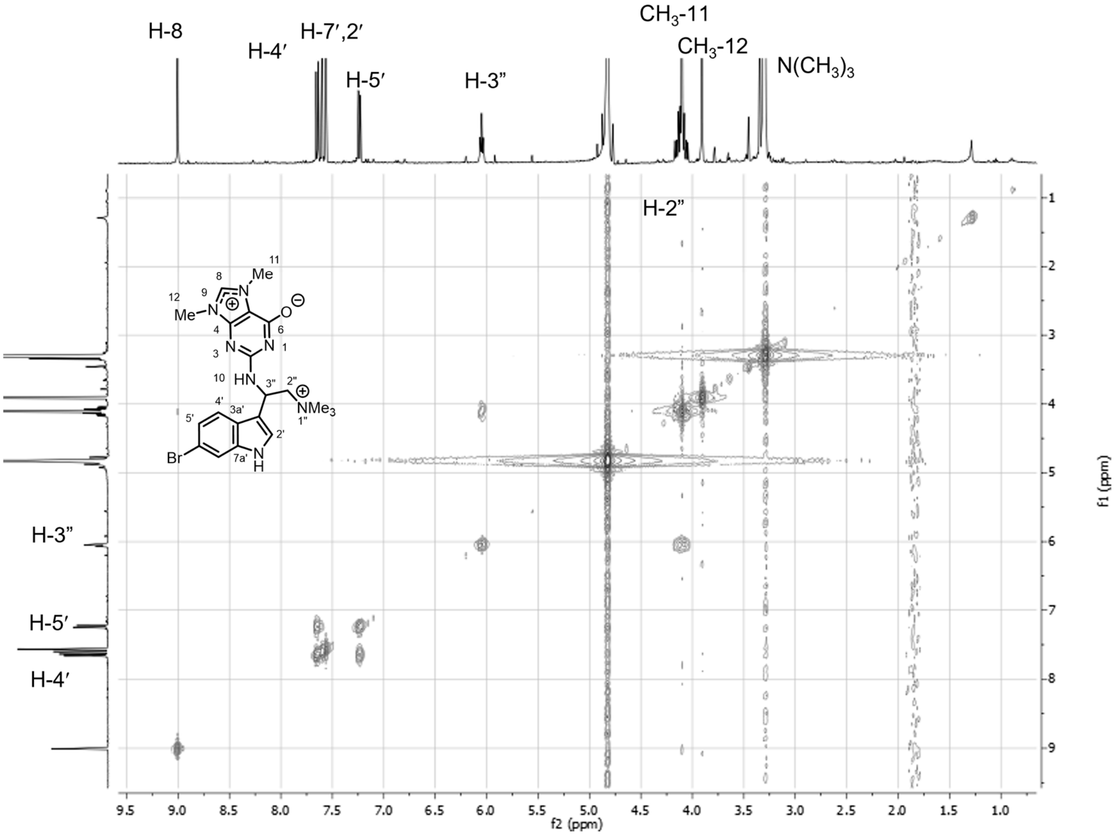

Figure S6. COSY spectrum of geobarrettin D (**1**).

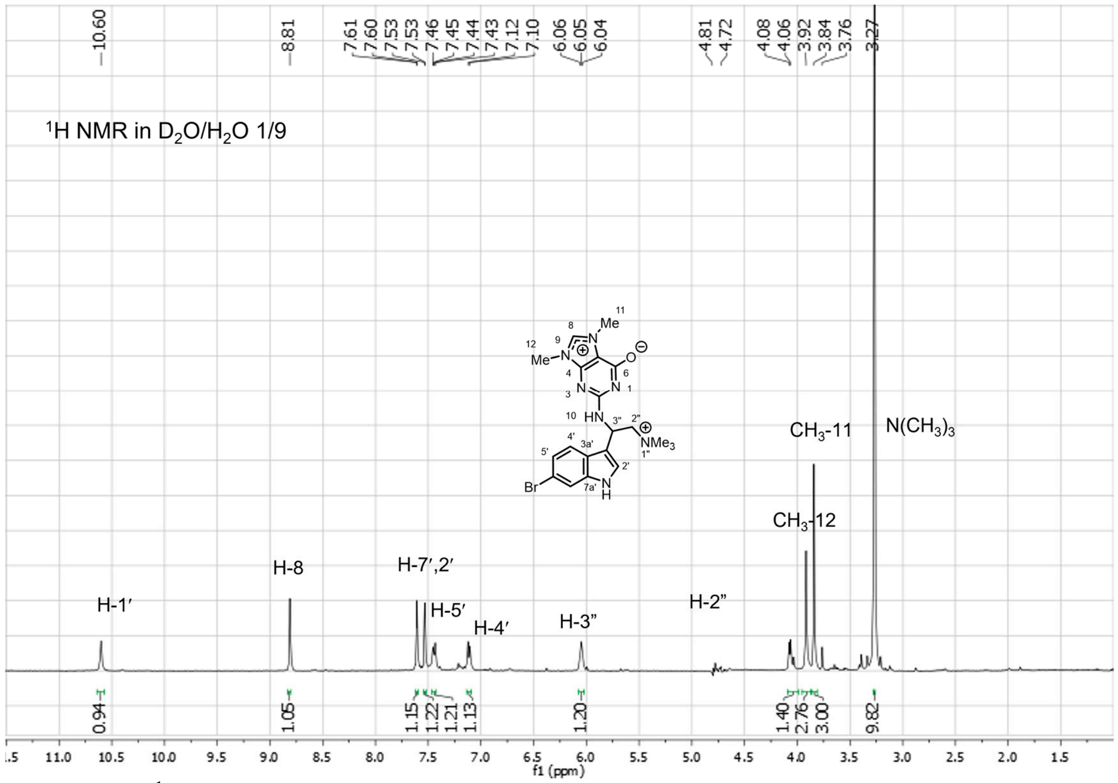

Figure S7. ^{1}H NMR spectrum of geobarrettin D (**1**), recorded in D_2O/H_2O 10/90, 600 MHz.

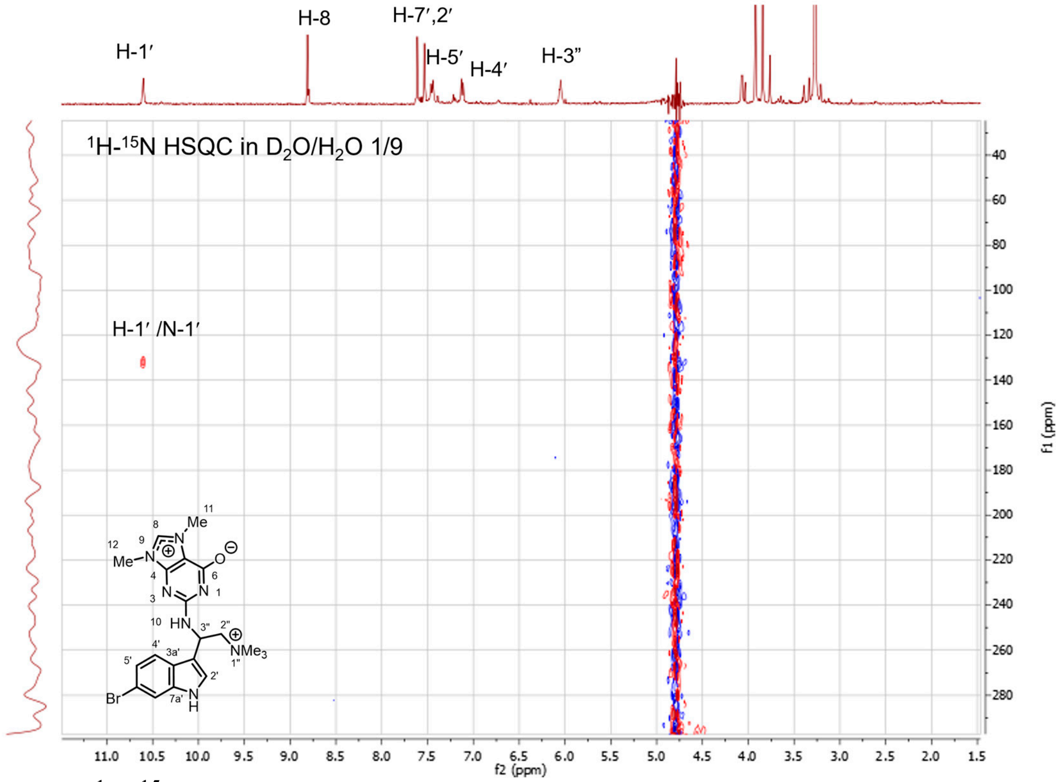

Figure S8. ^{1}H-^{15}N HSQC spectrum of geobarrettin D (**1**), recorded in D_2O/H_2O 10/90.

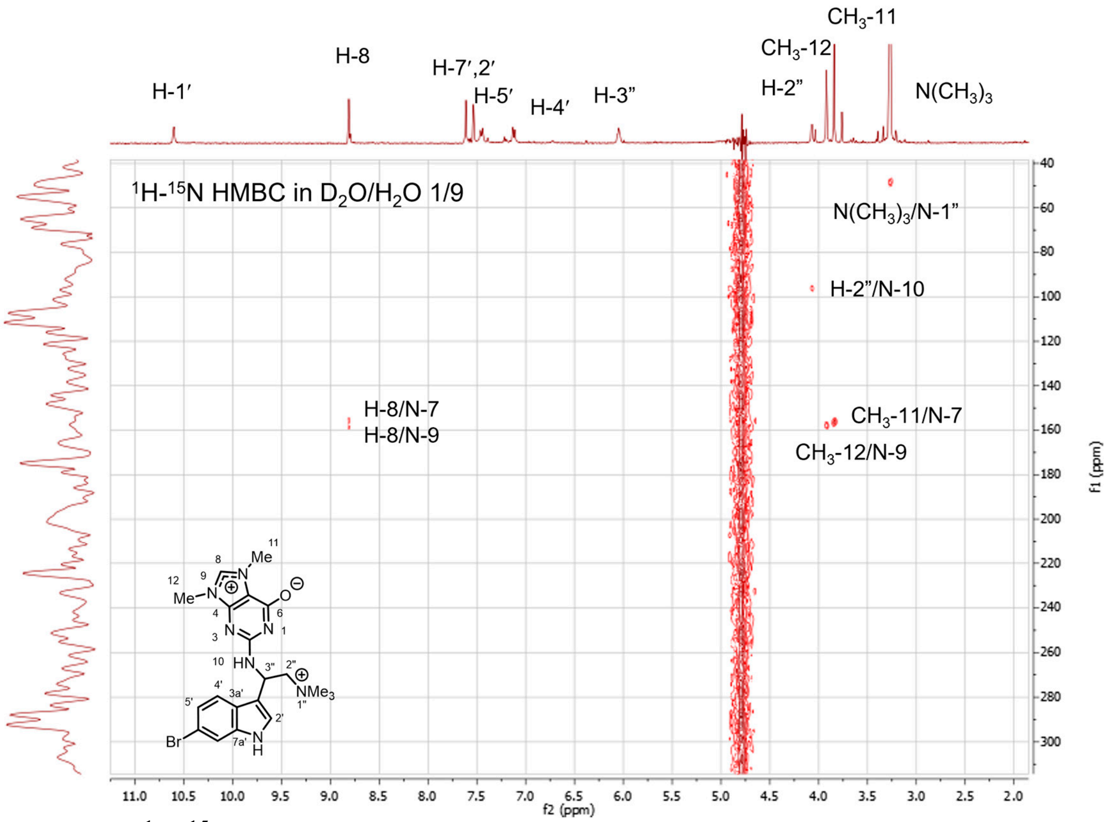

Figure S9. ^{1}H-^{15}N HMBC spectrum of geobarrettin D (**1**), recorded in D_2O/H_2O 10/90.

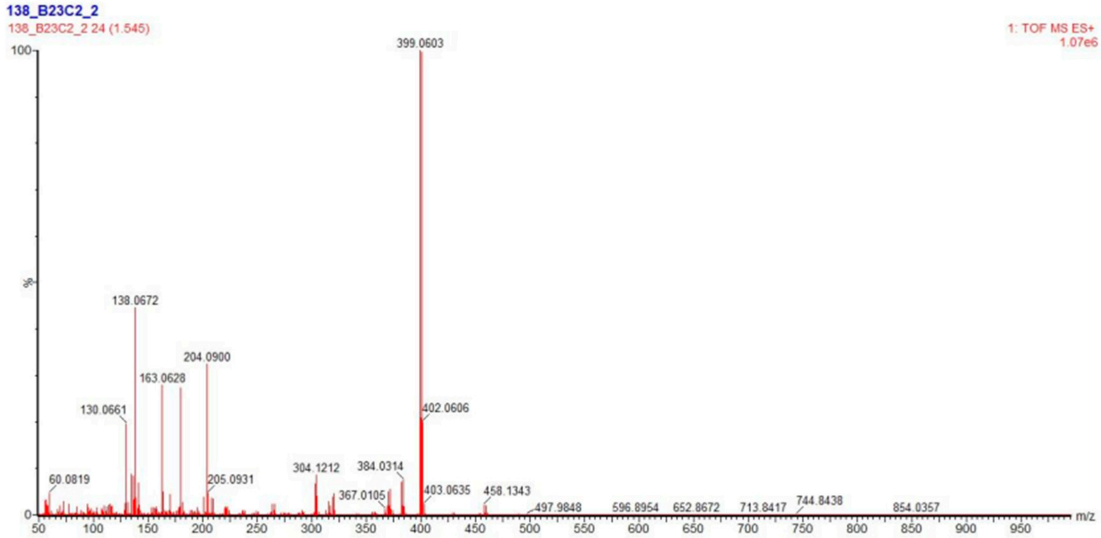

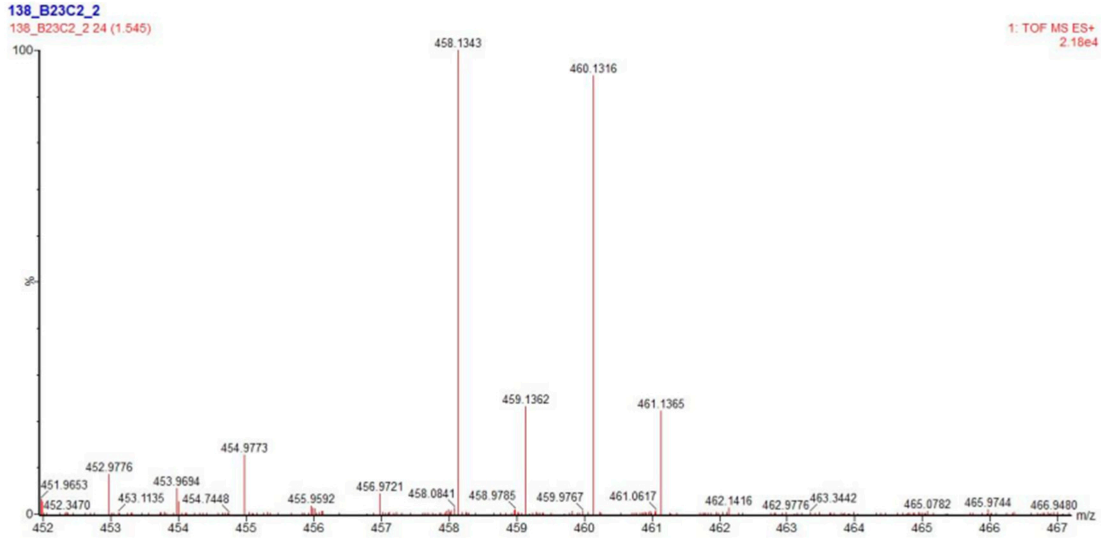

Figure S10. ESI spectrum of geobarrettin D (**1**)

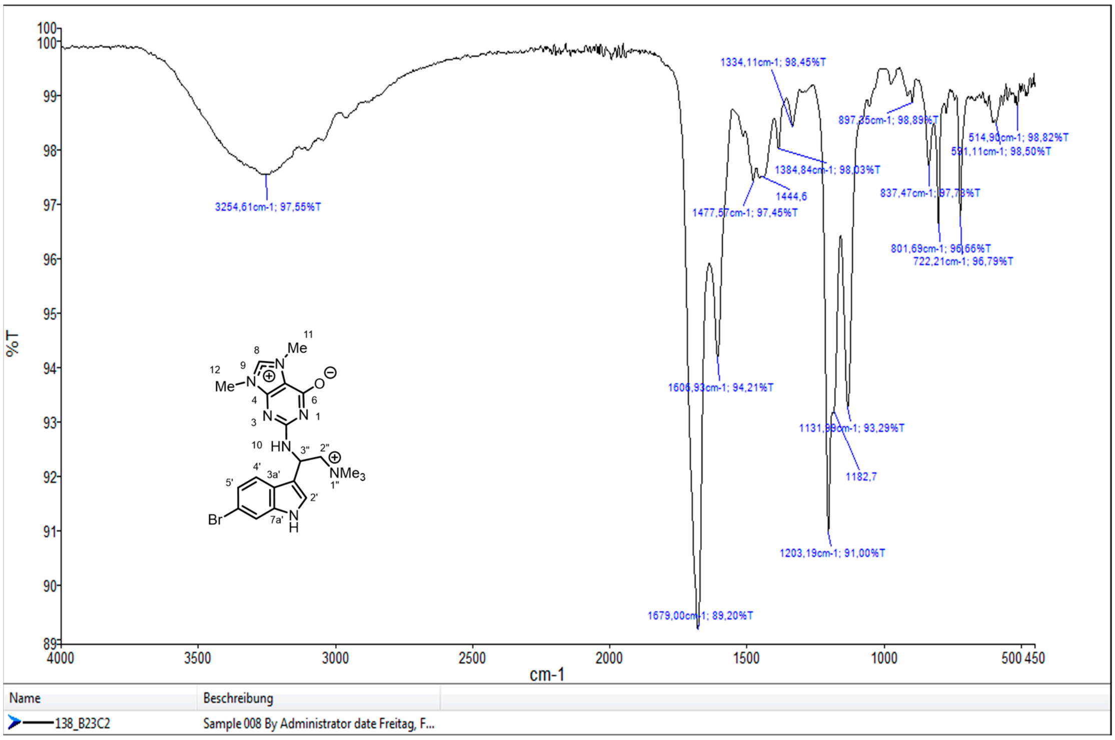

Figure S11. IR spectrum of geobarrettin D (**1**)
